# Supplementary material for: Chromothripsis during telomere crisis is independent of NHEJ, and consistent with a replicative origin
Source: Genome Res. 2019 May;29(5):737–49. doi: 10.1101/gr.240705.118 (PMC6499312; doi:10.1101/gr.240705.118)
Supplement: Supplemental Material [file supp_gr.240705.118_Supplemental_file_1.zip › contigs/annotated_contigs/DB57/contig.2.DB57_length_381_mean_cov_3.95538057743.docx]

**DB57_length_381_mean_cov_3.95538057743**

```
GCTGGAGGAGCTGGTTTAAAATACTGTATATAATCATATCTTTATGTTTTGTCAAAAATTATTGGTTTTAAAGAGAATATTTTATACAT
 >chr7:127148748-127149010 - E=3e-125
GTTATTTTGTTGTCAAAAATATGATAAAATAGTATGAATATTATTGCACACATATCTTGAAAACTATGAACGCATAATTTCAAGAACCT

GAGTGATGTATCTATTCTCCATAATTGTAGCTTGATTGTAGATCACCAAACAGGAATAAGCCTCCAAAAGTAAAGAAGATACTA|CTGT
                                                                                      >c
AGCTCCTATGCTCTTTCTACAGTAGAGAAGGGCTTCAGAGAGGACAGGATGGATGACGTCAGCCCCCCCGATAAAGGAGGGTGTGAG|A
hr7:134290367-134290458 + E=3e-34
TCACGTGCGGCAGTTGGGGGAGGGGAA
```
